# Erectile dysfunction pre and post kidney transplant recipients in Duhok city; cross sectional study

**DOI:** 10.1016/j.amsu.2020.04.038

**Published:** 2020-05-16

**Authors:** Shakir Saleem Jabali, Zana Sidiq M. Saleem, Ayad Ahmad Mohammed, Newar M. Mahmood

**Affiliations:** a Urologist, Department of Surgery, College of Medicine, University of Duhok, Kurdistan Region, Iraq; b Nephrologist, Department of Medicine, College of Medicine, University of Duhok, Kurdistan Region, Iraq; c General Surgeon, Department of Surgery, College of Medicine, University of Duhok, Kurdistan Region, Iraq; d Urologist, Duhok Directorate General of Health, Kurdistan Region, Iraq

**Keywords:** Erectile dysfunction, Renal transplantation, Sexual function, Chronic kidney diseases, IIEF-5

## Abstract

**Introduction:**

Sexual dysfunction is a common problem in patients with chronic kidney disease. Disturbances in sexual function are noticed in early stages of chronic kidney diseases and deteriorate further as renal function declines. This is due to uremic effects, comorbid illness, anemia, hormonal disturbances, autonomic neuropathy, vascular diseases, hyperparathyroidism, hyperprolactinemia, side effects of medications, and psychosocial factors.

**Patients and method:**

This is a cross-sectional study which included 59 male patients who underwent renal transplantation for more than 6 months. The International Index of Erectile Function (IIEF-5) was adopted in our study to record the erectile function.

**Results:**

The mean age was 49.41 years, and the mean number of hemodialysis per month was 5.31. The cause of the chronic kidney disease was diabetes mellitus in 35.59%, glomerulonephritis in 20.34%, and hypertension in 16.95%, other causes were diagnosed in order of decreasing frequency. Most patients developed improvement in the erectile function after transplantation. There was significant correlation with 3 of the elements of the IIFE-5.i.e; penile hardness pre-penetration, Maintaining erection during intercourse, and Difficulty to maintain erection to complete the intercourse (p values 0.015, 0.011, and 0.023) respectively, and the overall improvement after transplantation which showed a p-value of less than 0.031, while there was no significant correlation with Confidence with erection and Satisfaction with intercourse before and after transplantation (p values 0.113 and 0.121) respectively. One of the patients (1.7%) developed severe dysfunction after that.

**Conclusion:**

ED is common sequel of chronic kidney disease. The etiology is multifactorial and may be worsen by advanced age, presence of diabetes mellitus and prolonged duration of hemodialysis. Renal transplantation has a positive impact on sexual function and lead to improvement of erectile dysfunction. Erectile dysfunction that persists after kidney transplantation is usually attributed to multiple preexisting comorbidities.

## Highlights

• Sexual dysfunction is a common problem in patients with chronic kidney disease.
• The etiology of Sexual dysfunction is multifactorial.
• Renal transplantation has a positive impact on sexual function.

## 1 Introduction

Sexual dysfunction (SD) is a common problem in patients with chronic kidney disease (CKD). Disturbances in sexual function are noticed in early stages of CKD and deteriorate further as renal function declines [1,2].

Sexual dysfunction has a major impact on the quality of life and it had been reported in both male and female patients with CKD. This greatly affects the social and the marital life of the affected patients [[3], [4], [5], [6]].

Seventy percent of males have some forms of erectile dysfunctions (ED) during their life time. Other reported sexual problems may include reduced libido, difficulty in achieving orgasm and anejaculation [7].

The etiology of SD is often multifactorial, besides the uremic effects, factors such as comorbid illness, anemia, hormonal disturbances, autonomic neuropathy, vascular diseases, hyperparathyroidism, hyperprolactinemia, side effects of medications, and psychosocial factors all contribute to the existence of SD [1,2].

Renal transplantation prolongs life and improves the quality of life in patients with CKD. Because of normalization of the hormonal disturbances, renal transplantation improves the sexual health (e.g. libido), energy, and fertility [[8], [9], [10], [11]].

However, after kidney transplantation, the prevalence of SD still remains 46% in both men and women. The persistence of SD after receiving a kidney transplant may have a negative impact on the patients’ well-being [[12], [13], [14], [15]].

### 1.1 Research registration

Ethical committee approval granted from the Duhok Directorate of General Health, Scientific Research Division, and email: scientific.research@duhokhealth.org.

The research is registered according the World Medical Association's Declaration of Helsinki 2013 at the research registry at the 13th of December 2019, Research registry UIN: research registry **5277.**

### 1.2 Aim of the study

The aim of the study is to assess the erectile dysfunction in male patients before and after kidney transplantation by means of a validated, self-administered questionnaire.

### 1.3 Patients and method

This is a cross-sectional study which included 68 male patients who underwent renal transplantation for more than 6 months, patients with stable graft function were included. All were male patients aged 40 years or older and who attended regular visits to the transplantation clinic in Duhok kidney transplantation center from January 2018 to march 2019 were considered eligible and were included in this study.

A translated questionnaire to the native local language from the original version of five items of the International Index of Erectile Function (IIEF-5) which was adopted in our study.

Patients were given the questionnaire to answer it at home, data were collected before the transplantation and after transplantation.

### 1.4 Definitions

ED is defined as the inability to achieve or maintain an erection sufficient to allow satisfactory sexual intercourse. Scoring for each item was done from 0 to 5. The total score ranges from 0 to 25 points, and the cutoff points proposed by the authors are as follow: severe dysfunction when the score ranges between 0 and 7, moderate dysfunction when the score ranges between 8 and 11, mild-to-moderate dysfunction score ranges between 12 and 16, mild dysfunction between 17 and 21, and no dysfunction between 22 and 25. Erectile dysfunction is defined as an IIEF-5 score of less than 22.

The statistical calculations were done in Statistical Package for Social Sciences (SPSS 25:00 IBM: USA).

The descriptive purposes of our study is displayed in frequency and percentages for categorical data and mean and standard deviation for continuous variables.

Correlation were done using the two-tailed t-tests with 95% confidence interval, P values less than 0.05 were considered significant.

The work of this article has been reported in line with the STROCSS criteria [16].

## 2 Results

Our study included 59 kidney transplant male patients, their mean age was 49.41 years, and the mean number of hemodialysis per month was 5.31, Table 1.

**Table 1:** Showing the characteristics of patients who were included in the study.

Main category	Subcategories	Frequency	Percentage
Age in years, M; SD Range 40 - 67		49.41	7.127
Residency	Urban Rural	34 25	57.6 42.4
Duration of hemodialysis in months, M; SD Range: 1–36 months		5.31	7.152
Number of hemodialysis per week, M; SD		1.54	1.039
Medications	PCP SCP ECP	26 32 1	44.1 54.2 1.7
GFR, M; SD	GFR before dialysis GFR after dialysis	7.07 72.17	3.713 25.323

Abbreviations: P: prednisolone, C: Cellcept (mycophenolate mofetil), P: prograf (tavrolimus), E: Everolimus, GFR: glomerular filtration rate.

**Fig. 1:**
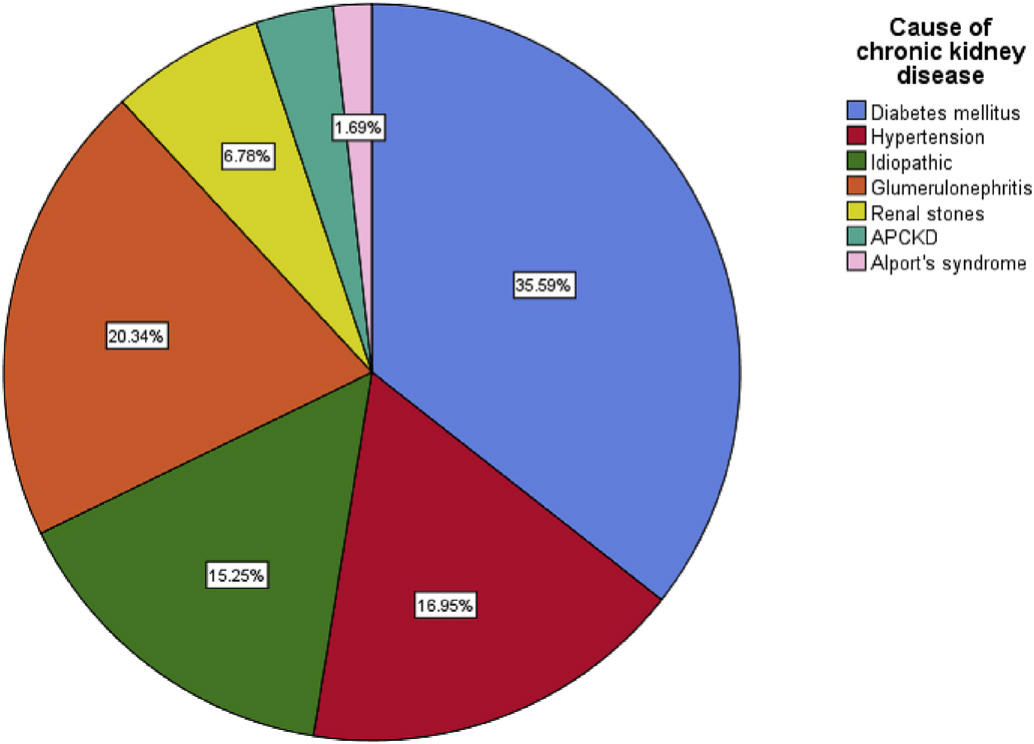
A simple pie chart showing the causes of chronic kidney disease which mandated renal transplantation.

The most cause of the chronic kidney disease was diabetes mellitus in 35.59%, followed by glomerulonephritis in 20.34%, hypertension in 16.95% of patients, and other causes were diagnosed in order of decreasing frequency, Fig. 1.

**Fig. 2:**
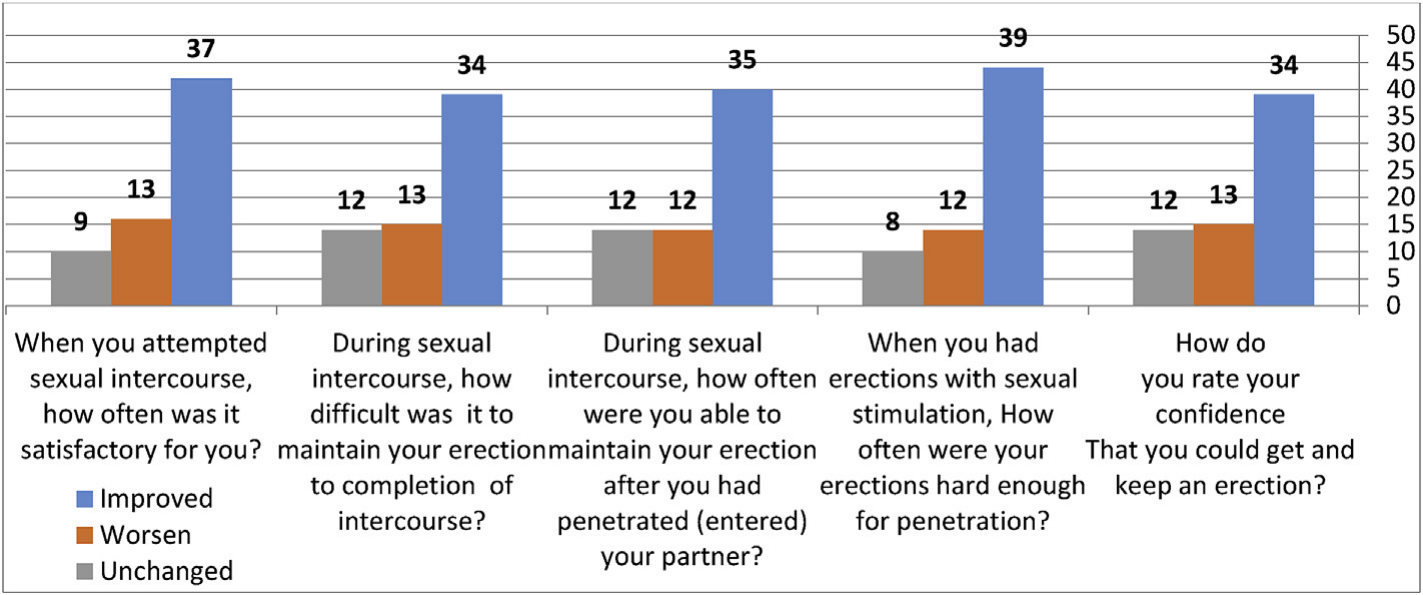
A bar chart showing the response from the patients to the IIEF-5 after renal transplantation.

**Fig. 3:**
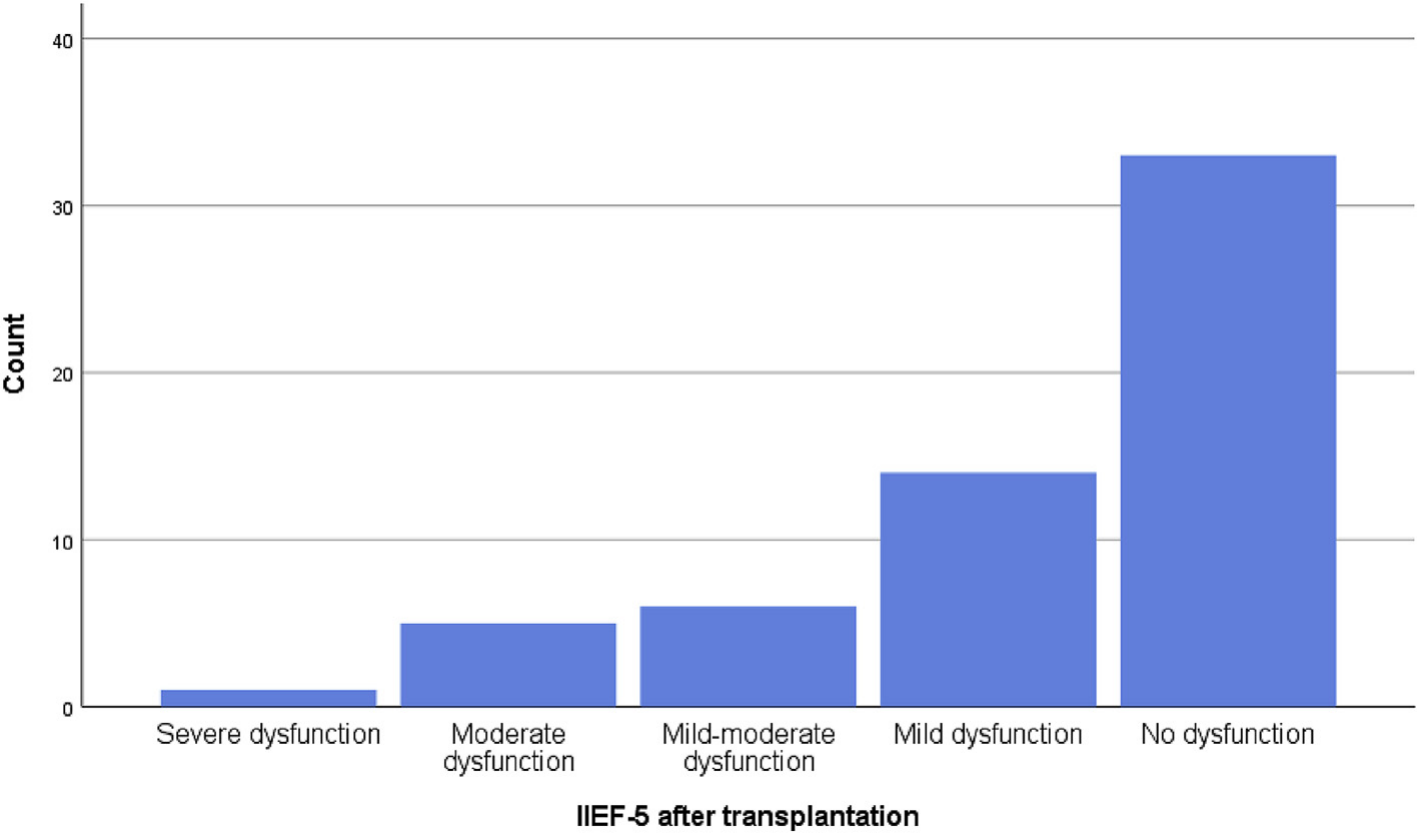
A simple bar chart showing the result of the erectile function after renal transplantation according the IIEF-5.

Most patients developed improvement in the erectile function after transplantation, there was a significant correlation with three of the elements of the IIFE-5 and the overall improvement after transplantation, Fig. 2 and 3, and Table 2.

**Table 2:** Showing the relation of different patterns of sexual performance in the involved patients before and after renal transplantation.

Erectile function, (n = 59)	Std. Error Mean	95% Confidence Interval of the Difference		Sig. (2-tailed)
		Lower	Upper	
Confidence with erection Before transplantation After transplantation	.158	-.570	.062	.113
Penile hardness pre-penetration Before transplantation After transplantation	.162	-.732	-.082	.015
Maintaining erection during intercourse Before transplantation After transplantation	.155	-.717	-.096	.011
Difficulty to maintain erection to complete the intercourse Before transplantation After transplantation	.175	-.756	-.057	.023
Satisfaction with intercourse Before transplantation After transplantation	.194	-.693	.083	.121
Overall IIEF-5 score	.787	−3.322	-.170	.031

The majority of patients had mild dysfunction before transplantation according to the IIEF-5 score, while after transplantation the majority of them reported a noticeable improvement, while one of the patients developed severe dysfunction, Table 3 & Fig. 2.

**Table 3:** Showing the IIEF-5 before and after renal transplantation.

IIEF-5	Subcategory	Frequency	Percentage
Before transplantation	Moderate dysfunction	2	3.4
	Mild-to-moderate dysfunction	15	25.42
	Mild dysfunction	35	59.32
	No dysfunction	7	11.86
After transplantation	Severe dysfunction	1	1.7
	Moderate dysfunction	5	8.47
	Mild-to-moderate dysfunction	6	10.16
	Mild dysfunction	14	23.72
	No dysfunction	33	55.95

## 3 Discussion

Erectile dysfunction is defined by the National Institute of Health (NIH) Consensus Development Conference as the inability to achieve or maintain an erection sufficient for satisfactory sexual performance. Although laboratory based diagnostic procedures are available, it has been proposed that sexual function is best assessed in a naturalistic setting with patient self-reporting technique [17].

Renal failure is a major risk for erectile dysfunction and about half of patients with chronic renal failure may be impotent which is multifactorial but primarily organic. In addition to the uremic milieu, peripheral neuropathy, autonomic insufficiency, peripheral vascular disease and pharmacotherapy, all play an important role in the development of erectile dysfunction [18].

Our study showed that ED is more common and more severe among patients aged 45 years and in those with history of hemodialysis. Increased age is a well-known risk factor for ED, the importance of increased age on the prevalence of ED in the renal transplant population has been verified in several studies, Malavaud et al., and his colleagues studied 271 kidney transplant patients, they found that the average age of those patients with ED was 49.2 years while those without ED was 44.5 years. They also found that ED is 3–4 times more likely to occur in those who are over 40 years old [[19], [20], [21]].

The most common cause of CKD in our study was diabetes mellitus, diabetes mellitus is also a major cause of ED. This is multifactorial and is thought to be caused by acceleration of angiopathy and neuropathy. Long duration of hemodialysis is also a major risk factor for ED. This has been attributed to permanent damage of cavernous vessels and smooth muscles sustained during the long course of renal failure [22,23].

Early transplantation is recommended because it is more effective with lower costs than hemodialysis. Maintaining erectile function should be another reason for early transplantation.

Regarding the role of kidney transplantation on sexual function, we found that majority of participant had improved ED post transplantation. There was significant correlation with 3 of the elements of the IIFE-5.i.e; penile hardness pre-penetration, Maintaining erection during intercourse, and Difficulty to maintain erection to complete the intercourse (p values 0.015, 0.011, and 0.023) respectively, and the overall improvement after transplantation which showed a p-value of less than 0.031, while there was no significant correlation with Confidence with erection and Satisfaction with intercourse before and after transplantation (p values 0.113 and 0.121) respectively. One of the patients (1.7%) developed severe dysfunction after that. Improvement of sexual function after kidney transplantation has been reported in several similar studies supporting our results [[24], [25], [26], [27], [28]].

In another more recent study of CKD patients receiving a spectrum of treatment such as peritoneal, hemodialysis, and renal transplant; renal transplant patients showed the most statistically significant increase in IIEF score [26,28].

In addition to the normalization of hormonal and metabolic functions post-transplant, there is a significant improvement of the psychosocial parameters in transplant recipients, which have a major impact on the sexual function as well. It is important to note that many patients have a persistent ED post-transplant, this is probably due to the fact that transplant alone cannot eliminate all comorbidities affecting both renal and erectile functions [21,29,30].

There was no significant association between ED and the use of different types of medications in this study, this result was comparable to other study in this regard [31].

## Source of funding

The author is the source of the funding.

## Provenance and peer review

Not commissioned, externally peer reviewed.

## Declaration of competing interest

No conflict of interest is present to be declared**.**

## References

[1] Rathi M., Ramachandran R. (2012). Sexual and gonadal dysfunction in chronic kidney disease: Pathophysiology. Indian J. Endocrinol. Metabol..

[2] Finkelstein F.O. (2007). Therapy Insight: sexual dysfunction in patients with chronic kidney disease. Nat. Rev. Nephrol..

[3] Muehrer R.J. (2009). Sexuality, an important component of the quality of life of the kidney transplant recipient. Transplant. Rev..

[4] Esen B. (2015). Evaluation of relationship between sexual functions, depression and quality of life in patients with chronic kidney disease at predialysis stage. Ren. Fail..

[5] Fernandes G.V. (2010). The impact of erectile dysfunction on the quality of life of men undergoing hemodialysis and its association with depression. J. Sex. Med..

[6] Raggi M.C. (2012). Sexual and relationship functioning before and after renal transplantation: a descriptive study with patients and partners. Scand. J. Urol. Nephrol..

[7] Antonucci M. (2015). Male sexual dysfunction in patients with chronic end-stage renal insufficiency and in renal transplant recipients. Arch. Ital. Urol. Androl..

[8] Muehrer R.J., Becker B.N. (2005). Psychosocial factors in patients with chronic kidney disease: life after transplantation: new transitions in quality of life and psychological distress. Seminars in Dialysis.

[9] Schulz T. (2014). Great expectations? Pre‐transplant quality of life expectations and distress after kidney transplantation: a prospective study. Br. J. Health Psychol..

[10] Filocamo M.T. (2009). Sexual dysfunction in women during dialysis and after renal transplantation. J. Sex. Med..

[11] Wang G.-c. (2010). Measurements of serum pituitary-gonadal hormones and investigation of sexual and reproductive functions in kidney transplant recipients. Int. J. Nephrol..

[12] Diemont W.L. (2000). Sexual dysfunction after renal replacement therapy. Am. J. Kidney Dis..

[13] Hricik D.E. (2001). Life satisfaction in renal transplant recipients: preliminary results from the transplant learning center. Am. J. Kidney Dis..

[14] Matas A.J. (2002). Life satisfaction and adverse effects in renal transplant recipients: a longitudinal analysis. Clin. Transplant..

[15] Muehrer R.J. (2014). Sexual concerns among kidney transplant recipients. Clin. Transplant..

[16] Agha R. (2019). STROCSS 2019 Guideline: strengthening the reporting of cohort studies in surgery. Int. J. Surg..

[17] Lue T.F. (1998). Evaluation and nonsurgical management of erectile dysfunction and priapism. Campbell Urol..

[18] Bellinghieri G. (2008). Sexual dysfunction in chronic renal failure. J. Nephrol..

[19] Mekki M. (2013). Prevalence and associated risk factors of male erectile dysfunction among patients on hemodialysis and kidney transplant recipients: a cross-sectional survey from Sudan. Saudi J. Kidney Dis. Trans..

[20] Malavaud B. (2000). High prevalence of erectile dysfunction after renal transplantation. Transplantation.

[21] El-Bahnasawy M. (2004). Critical evaluation of the factors influencing erectile function after renal transplantation. Int. J. Impot. Res..

[22] Costabile R.A. (2003). Optimizing treatment for diabetes mellitus induced erectile dysfunction. J. Urol..

[23] Kaufman J.M. (1994). Impotence and chronic renal failure: a study of the hemodynamic pathophysiology. J. Urol..

[24] Laupacis A. (1996). A study of the quality of life and cost-utility of renal transplantation. Kidney Int..

[25] Soykan A. (2005). Do sexual dysfunctions get better during dialysis? Results of a six-month prospective follow-up study from Turkey. Int. J. Impot. Res..

[26] Nassir A. (2009). Sexual function in male patients undergoing treatment for renal failure: a prospective view. J. Sex. Med..

[27] Shamsa A., Motavalli S.M., Aghdam B. (2005). Erectile function in end-stage renal disease before and after renal transplantation. Transplantation Proceedings.

[28] Ahmad M. (2009). Impact of renal transplantation on erectile dysfunction due to chronic renal failure in male patients. J. Ayub Med. Coll. Abbottabad.

[29] Charmet G.P. (1990). Sexual function in dialysis patients. Psychological and Physiological Aspects of Chronic Renal Failure.

[30] Mirone V. (2009). Renal transplantation does not improve erectile function in hemodialysed patients. Eur. Urol..

[31] Tripathi D. (2003). Erectile dysfunction after renal transplantation: a cross sectional study in northern India. Indian J. Nephrol..

